# Anomalous dispersion analysis of inhibitor flexibility: a case study of the kinase inhibitor H-89

**DOI:** 10.1107/S1744309112028655

**Published:** 2012-07-26

**Authors:** Alexander Pflug, Kenneth A. Johnson, Richard A. Engh

**Affiliations:** a Norwegian Structural Biology Centre, Department of Chemistry, University of Tromsø, N-9037 Tromsø, Norway

**Keywords:** protein kinase A, kinases, ligands, inhibitors, flexibility, anomalous dispersion, SAD, bromine

## Abstract

With its ability to show the interactions between drug-target proteins and small-molecule ligands, X-ray crystallography is an essential tool in drug-discovery programmes. However, its usefulness can be limited by crystallization artifacts or by the data resolution, and in particular when assumptions of unimodal binding (and isotropic motion) do not apply. Discrepancies between the modelled crystal structure and the physiological range of structures generally prevent quantitative estimation of binding energies. Improved crystal structure resolution will often not aid energy estimation because the conditions which provide the highest rigidity and resolution are not likely to reflect physiological conditions. Instead, strategies must be employed to measure and model flexibility and multiple binding modes to supplement crystallographic information. One useful tool is the use of anomalous dispersion for small molecules that contain suitable atoms. Here, an analysis of the binding of the kinase inhibitor H-89 to protein kinase A (PKA) is presented. H-89 contains a bromobenzene moiety that apparently binds with multiple conformations in the kinase ATP pocket. Using anomalous dispersion methods, it was possible to resolve these conformations into two distinct binding geometries.

## Abstract 2

The anomalous dispersion signal of the bromine-containing kinase inhibitor H-89 was used to characterize discrete binding modes of the compound when complexed with the catalytic subunit of protein kinase A.

## 1. Introduction   

**Figure 1:**
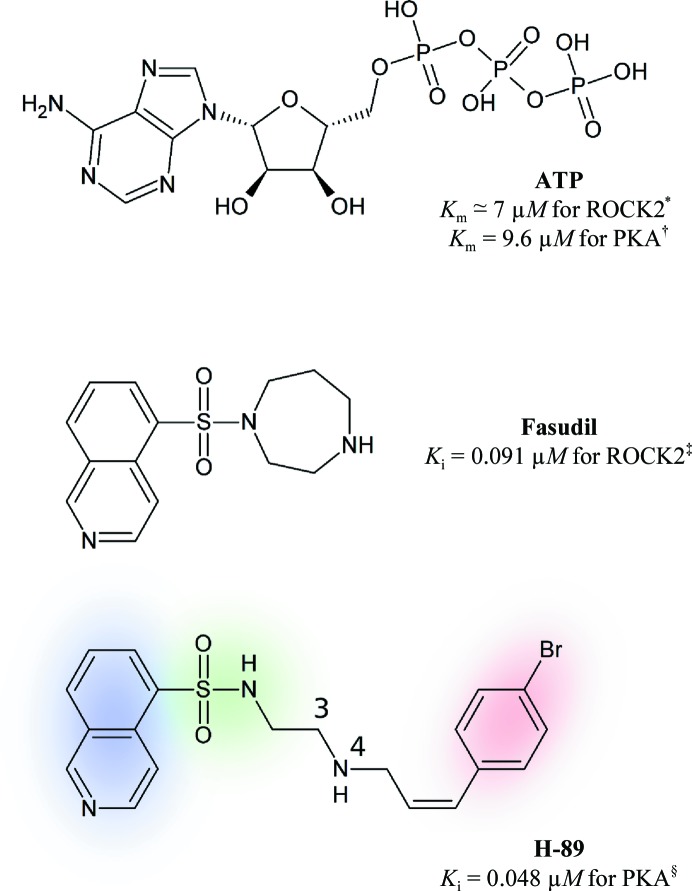
Chemical structures of adenosine-5′-triphosphate (ATP), Fasudil and H-89 in similar orientations with respect to the ATP pocket (hinge region at the left). The isoquinoline portion of H-89 is highlighted in blue, the sulfonamide portion in green and the bromobenzene moiety in red. The numbering of the H-89 molecule refers to C3 and N4, consistent with the designation of the atoms in the structure 3vqh. ‘PKA’ refers to cAMP-dependent protein kinase catalytic subunit α isoform 1 and ‘ROCK2’ to Rho kinase α. Affinity values were taken from the literature: *, Rajagopalan *et al.* (2010 ▶); †, Gassel *et al.* (2003 ▶); ‡, Yano *et al.* (2008 ▶); §, Engh *et al.* (1996 ▶).

Apart from the approved drug Fasudil (HA-1077), H-89 is one of the most prominent representatives of the ‘H-series’ of kinase inhibitors, a set of ATP-competitive isoquinoline sulfonamides (Chijiwa *et al.*, 1990 ▶; Hidaka *et al.*, 1984 ▶; Ono-Saito *et al.*, 1999 ▶; Fig. 1 ▶). H-89 was developed and reported to be selective towards the catalytic subunit of cAMP-dependent protein kinase, also known as protein kinase A (PKA). Despite its misregulation in certain types of cancer, PKA is usually considered to be an ‘antitarget’ in drug development owing to the ubiquitous and essential nature of the cellular processes that it regulates. Hence, the use of H-89 has largely remained confined to academic research. In contrast, the Rho kinase-targeting inhibitor Fasudil was approved in Japan in 1995 for the prevention of cerebral vasospasm in patients with subarachnoid haemorrhage and was found to potentially be useful to enhance the memory and improve the prognosis of Alzheimers patients (Huentelman *et al.*, 2009 ▶). However, H-89 became particularly popular for *in vitro* studies requiring the absence of PKA activity or on the regulatory role of PKA itself. It is still used frequently, but now in the context of recent studies that have shown H-89 to be a rather general AGC kinase inhibitor (Davies *et al.*, 2000 ▶; Lochner & Moolman, 2006 ▶). While one barrier to the development of H-series compounds as drugs may be the inhibition of PKA, H-89 has also proven to be useful in drug-design projects. The H-­89 scaffold has provided the basis for the design of new compounds with selectivity towards protein kinase B (PKB/Akt; Caldwell *et al.*, 2008 ▶; Collins *et al.*, 2006 ▶; Reuveni *et al.*, 2002 ▶), which is structurally similar to PKA (Gassel *et al.*, 2003 ▶) and remains an important drug target (Cheng *et al.*, 2005 ▶; Wu & Hu, 2010 ▶).

## 2. Materials and methods   

### 2.1. Protein production, purification and crystallization   

The full-length human catalytic subunit α of PKA (GenBank accession No. NP_002721) was expressed in *Escherichia coli* BL21 (DE3)-RIL cells (Stratagene) from a construct based on the vector pT7-7 in auto-induction medium (Studier, 2005 ▶) over a period of approximately 20 h at 297 K. The procedures used for the purification of PKA followed previously published protocols (Engh *et al.*, 1996 ▶).

Cocrystallization of PKA and H-89 was carried out in hanging drops at 277 K. Drops consisting of 10 mg ml^−1^ protein, 25 m*M* bis-tris/MES pH 6.9, 50 m*M* KCl, 1.5 m*M* octanoyl-*N*-methylglucamide, 1 m*M* ‘protein kinase inhibitor’ peptide (PKI; _5_TTYADFIASGR­TGRRNAIHD_24_) and 5 m*M* H-89 (added from a 100 m*M* methanol stock) were equilibrated against 12–22%(*v*/*v*) methanol. For data collection, crystals were transferred into 30% 2-methyl-2,4-pentanediol and flash-cooled.

### 2.2. Diffraction data collection and data processing   

The diffraction of a cooled crystal was measured on beamline ID29 at the European Synchrotron Radiation Facility (ESRF; Grenoble, France). The wavelength of 0.91969 Å was chosen for data collection after a scan for the maximum X-ray absorption of the crystal in proximity to the theoretical *K* absorption edge of bromine, and the data-collection strategy was designed to obtain an overall multiplicity of greater than four. Subsequent processing of the data was carried out with the *XDS* software package (Kabsch, 2010 ▶) and the *CCP*4 program suite (Winn *et al.*, 2011 ▶). The diffraction frames were integrated with *XDS* and the resulting intensities were scaled with *XSCALE*, in which Friedel pairs were not merged (‘FRIEDEL’S_LAW=FALSE’ option; Table 1 ▶). The data set was phased by molecular replacement with *MOLREP* (Vagin & Teplyakov, 2010 ▶) employing the coordinates of PDB entry 1ydt (Engh *et al.*, 1996 ▶). The structure was refined with *REFMAC*5 (Murshudov *et al.*, 2011 ▶; Table 1 ▶) and the resulting structure factors were merged with the columns ‘DANO’ and ‘SIGDANO’ of the unphased original *.mtz file using the program *CAD*. The resulting *.mtz file containing both the structure factors with phases and the anomalous signal of the bromine of H-89 was used to calculate anomalous difference Fourier maps with the program *FFT* (Ten Eyck, 1973 ▶).

**Figure 2:**
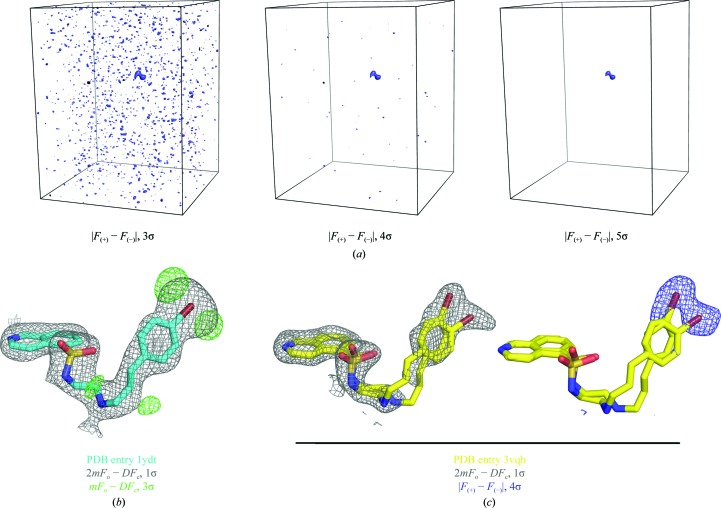
(*a*) Anomalous difference Fourier maps (1.95 Å) generated to cover all atoms of the asymmetric unit in PDB entry 3vqh contoured at levels of 3σ, 4σ and 5σ. (*b*) 2.3 Å resolution electron-density map (grey) and difference density map (green) surrounding the compound H-89 in PDB entry 1ydt (cyan; Engh *et al.*, 1996 ▶). (*c*) 1.95 Å resolution OMIT electron-density map (grey) and anomalous difference density map (blue) carved around the two conformations of compound H-89 in PDB entry 3vqh (yellow).

*REFMAC*5 (Murshudov *et al.*, 2011 ▶) was used to generate weighted electron-density and difference maps (2*mF*_o_ − *DF*_c_ and *mF*_o_ − *DF*_c_, respectively) for the refined structures (Figs. 2 ▶ *b* and 2 ▶ *c*). *F*_o_ and *F*_c_ refer to the observed and model structure factors, *m* is the figure of merit and *D* is the model error parameter. The *REFMAC*5 calculation of the weighting factors *D* and *m* (Murshudov *et al.*, 2011 ▶) matches that in *SIGMAA* (Read, 1986 ▶) except that only the free-*R*-­flagged reflections are used to estimate *m* and *D*. For missing reflections, the map coefficients are replaced with *DF*_c_ in electron-density maps and zero in difference maps.

### 2.3. Inhibitors   

The kinase inhibitor H-89 was purchased from Cayman Chemicals (Ann Arbor, USA). The ‘protein kinase inhibitor’ peptide (PKI; _5_TTYADFIASGRTGRRNAIHD_24_) used in the purification and cocrystallization of PKA was purchased from GL Biochem Shanghai Ltd (Shanghai, People’s Republic of China).

### 2.4. Structure deposition   

The coordinates and structure factors of the PKA–H-89 complex crystal structure described here have been deposited in the Protein Data Bank (PDB) with accession code 3vqh.

## 3. Results   

### 3.1. Overall structure   

**Figure 3:**
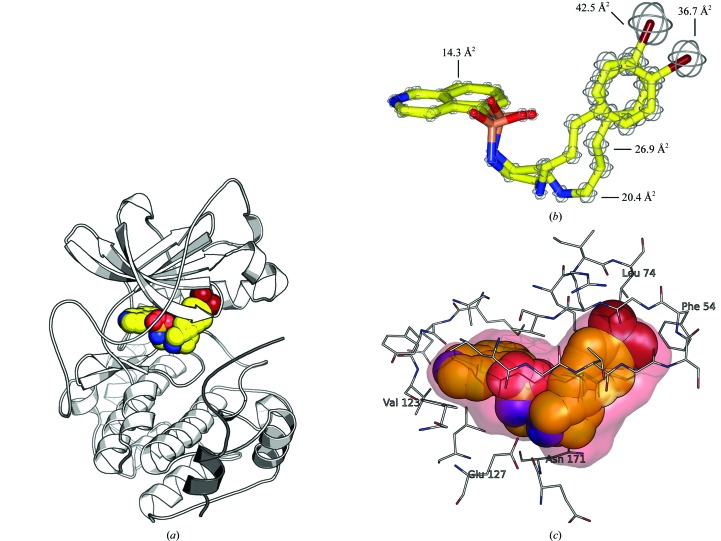
(*a*) Overall structure of PDB entry 3vqh: a ternary complex of the catalytic subunit α of protein kinase A (PKA; white), the peptidic pseudosubstrate ‘protein kinase inhibitor’ (PKI; grey) and the ATP-competitive inhibitor H-89 (yellow). (*b*) *B*/temperature factors of the H-89 conformers in the structure 3vqh plotted as spheres on the respective atoms. The values are indicated for selected atoms. (*c*) Binding environment of the H-89 conformers in the ATP pocket of PKA in structure 3vqh. Residues Val123, Glu127 and Asn171 form hydrogen bonds to the ligands; the bromine moieties pack against hydrophobic atoms from the side chains of Phe54 and Lys174 and some main-chain atoms of the glycine-rich loop. The inner surface of the ATP pocket is represented in red (maximum distance of 2 Å from the inhibitor molecule).

In PDB entry 3vqh the catalytic subunit α of protein kinase A appears in its usual conformation (Fig. 3 ▶ *a*), similar to that in reference structures such as 1atp (Zheng *et al.*, 1993 ▶) and 1cdk (Bossemeyer *et al.*, 1993 ▶) and nearly identical to an earlier structure of a PKA–H-89 complex (PDB entry 1ydt; Engh *et al.*, 1996 ▶). The root-mean-square deviation (r.m.s.d.) between the structures 1ydt and 3vqh is 0.33 Å. In 3vqh the fragment 5–24 of the PKI (‘protein kinase inhibitor’) peptide, which is routinely used for structural work on PKA as it stabilizes the kinase domain and facilitates crystallization, occupies the peptide-substrate site, which is formed primarily by the surface of the α-helical C-terminal lobe of the protein. The N-terminal lobe, which is mainly comprised of a five-stranded antiparallel β-sheet, is linked covalently to the C-terminal lobe by a single peptide chain (the hinge). Their interface forms a deep cleft which constitutes the binding pocket for the nucleotide substrate ATP. In the structure described here, the ATP-competitive inhibitor H-89 occupies the ATP-binding site (Fig. 3 ▶ *a*). As shown in Fig. 1 ▶, H-89 binds with respect to ATP such that the isoquinoline group of H-89 occupies the adenine-binding pocket, the sulfonamide mimics the ribose group of ATP and the bromobenzene moiety occupies the site of the phosphate groups beneath the glycine-rich loop (formed by β-strands 1 and 2 and the β-turn that links them).

As in PDB entry 1ydt (Engh *et al.*, 1996 ▶), the electron density for the bromobenzene portion of H-89 is diffuse and its position is not clearly defined (Fig. 2 ▶).

### 3.2. The anomalous signal describes a bivalent binding mode for H-­89   

The anomalous signal of the bromine of H-89 in the collected data set is rather weak, as is evident from the ‘Anomal. corr.’ and ‘SigAno’ parameters in Table 2 ▶, which represent the mean correlation factor between two random subsets of anomalous intensity differences and the mean anomalous difference in units of its estimated standard deviation, respectively. As would be hoped for a diffraction data set containing useful anomalous dispersion information, the anomalous correlation (‘Anomal. corr.’) exceeds 30% and the anomalous signal is stronger than noise (‘SigAno’ > 1), but this applies only for resolutions coarser than ∼5 Å (Table 2 ▶). However, although the strength of the anomalous signal is far below the requirements for a successful SAD phasing experiment (Dauter *et al.*, 2002 ▶), it is sufficient to unambiguously localize the position of the bromine moiety of H-89 within the asymmetric unit (Fig. 2 ▶).

The anomalous difference Fourier maps in Fig. 2 ▶(*a*) display a single strong feature in the asymmetric unit which is dominant in the maps contoured at signal-to-noise ratios of 3σ and 4σ and is unique in the map contoured at 5σ. This peak corresponds to the localization of the bromine group of H-89 in the ATP pocket of PKA (Fig. 2 ▶ *c*). However, the anomalous density is not localized to one site but appears spread out into two spheres of density, indicating two main positions of the bromine moiety. This result correlates well with the unclear electron density of the bromobenzene group of H-89 in both the structure 1ydt (Fig. 2 ▶ *b*; Engh *et al.*, 1996 ▶) and the higher resolution H-89–PKA complex structure determined in this study (Fig. 2 ▶ *c*). In both cases it seems that the bromobenzene group of H-89 has some freedom to rotate about an axis running perpendicular to its benzene ring. While the electron density alone hints at this, the anomalous density shows distinctly preferred positions of the Br atom.

Consistent with the appearance of two peaks of similar intensity in the anomalous difference Fourier map, the ligand H-89 was modelled in the structure 3vqh with two alternative conformations, each with 50% occupancy. Rotation of the C3—N4 bond (Fig. 1 ▶) in the flexible linker of H-89 and adjustment of the following dihedrals placed the bromine moieties of the two conformers into the distinct positions indicated by the anomalous difference map. The coordinates of the structure were subsequently refined in *REFMAC*5 without positional restraints for the ligand molecule, resulting in the conformations presented in Fig. 2 ▶(*c*). In the refinement the bromobenzene moieties of H-89 retained their distinct positions in good agreement with the density of the anomalous difference map. The linker geometries are correspondingly displaced relative to one another; this is in accord with the partial weak electron density of this portion of H-89 in the structure 3vqh (Fig. 2 ▶ *c*). In contrast, the isoquinoline moiety of H-89 is anchored to the hinge of the kinase domain *via* a hydrogen bond (Fig. 3 ▶ *c*) and hence features the lowest temperature factors in the ligand molecule (Fig. 3 ▶ *b*); the adjacent sulfonamide group shows a slight rotation. The torsion angles of the amide groups with respect to the isoquinoline vary by ∼17°. The protein–ligand interactions between PKA and the two conformers differ marginally. This is true for both the polar contacts of the linker of H-89 with PKA and the hydrophobic contacts between the bromobenzene moiety of H-89 and the glycine-rich loop of PKA (Fig. 3 ▶ *c*). In either case the bromine moiety of H-89 is not involved in polar contacts to the protein and the discrete positions of its two conformers are likely to be a consequence of constraints imposed by the linker dihedrals.

### 3.3. Data quality   

The diffraction data utilized for modelling the structure 3vqh originate from a single crystal which was exposed to an X-ray dose of approximately 7 MGy. Because neither the overall diffraction quality nor the anomalous signal decayed during data collection, the disorder of the bromine does not appear to result from radiation damage and both H-89 conformers are clearly observable. Consistently, the bromine moieties of both H-89 conformers appear electron-dense and well observed. The lengths of the bromine–carbon bonds in the bromobenzene moieties were both 1.9 Å, which is in good agreement with literature values.

## 4. Discussion   

The approach of incorporating bromine into small-molecule ligands in order to quickly screen for binding and to subsequently efficiently determine the binding geometry has been employed as a drug-discovery business model (Antonysamy *et al.*, 2008 ▶; Blaney *et al.*, 2006 ▶; Wolf *et al.*, 2002 ▶). Details of its application and utility are sparse in the scientific literature. However, this would be one approach to address the problem of evaluating ligand flexibility in drug design (Seddon *et al.*, 2012 ▶). Here, we show the successful use of this approach for a very specific application, namely the characterization of the apparently heterogeneous binding mode of a kinase inhibitor, which was not possible using electron-density maps (2*mF*_o_ − *DF*_c_) alone.

Although the statistics showed the overall anomalous signal to only be significant in lower resolution shells, the effects of the total signal transformed into the real-space anomalous electron-density map were unambiguously localized at the bromine group of H-89 with a resolution sufficient to identify two discrete positions. This demonstrates the utility of the approach for other related applications in characterizing binding-mode heterogeneity and also confirms its usefulness for applications involving weak-binding ligands or low-affinity small-molecule fragments that may bind with only partial occupancies.

Regarding the flexible binding mode of H-89 in the ATP pocket of PKA, the question arises whether this information may be useful for application in inhibitor design. In general, binding flexibility is associated with adaptability to variation in binding sites, consistent with the broad inhibition profile of H-89 for AGC kinases (Davies *et al.*, 2000 ▶; Lochner & Moolman, 2006 ▶). In order to develop H-89 towards a PKB inhibitor the linker between its aromatic moieties was rigidified, but the selectivity of the resulting compounds was not reported (Caldwell *et al.*, 2008 ▶; Collins *et al.*, 2006 ▶). An interesting approach could be to modify the linker of H-89 to capture the two respective binding conformations and investigate potential changes in the target-selectivity pattern of the compounds.

## Supplementary Material

PDB reference: PKA–H-89 complex, 3vqh

## Figures and Tables

**Table 1:** Refinement and structure statistics of PDB entry 3vqh Values in parentheses are for the last shell.

Data collection and scaling (*XDS*)	
X-ray source	ID29, ESRF
Resolution limits ()	35.01.95 (2.201.95)
Unit-cell parameters ()	*a* = 72.73, *b* = 75.18, *c* = 80.33
Space group	*P*2_1_2_1_2_1_
Wavelength ()	0.91969
Total No. of reflections	283804 (84880)
No. of unique reflections	61702 (18509)
Multiplicity	4.6 (4.6)
*I*/(*I*)	19.08 (5.68)
*R*_mrgd-*F*_ † (%)	9.0 (28.8)
Completeness (%)	98.0 (99.3)
Wilson *B* (^2^)	21.57
Refinement (*REFMAC*5)	
*R*_work_ ‡ (%)	19.0
*R*_free_ ‡ (%)	23.4
Average *B* factor (^2^)	21.88
No. of protein atoms	3015
No. of ligand atoms	62
No. of water molecules	178
R.m.s.d. bond lengths ()	0.010
R.m.s.d. bond angles ()	1.38
Ramachandran plot (*PROCHECK*)	
Most favoured (%)	91.1
Additionally allowed (%)	8.9
Generously allowed (%)	0
Disallowed (%)	0

† *R*_mrgd-*F*_ = 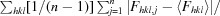 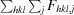.

‡ *R*_work_/*R*_free_ = 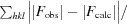 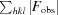.

**Table 2:** Selected columns from the *XSCALE* scaling statistics of PDB entry 3vqh

Resolution limit ()	Completeness of data (%)	*I*/(*I*)	*R*_mrgd-*F*_ (%)	Anomal. corr.† (%)	SigAno‡
35	0.0	99	99.9	0	0
30	90.0	65.84	1.3	35	1.870
25	90.9	32.47	2.4	3	1.318
20	96.7	62.23	1.4	75	1.661
15	100.0	56.57	1.3	57	1.599
10	100.0	59.68	1.2	47	1.132
9	100.0	54.80	1.3	53	1.224
8	100.0	53.60	1.6	42	1.211
7	100.0	50.58	1.6	44	1.457
6	100.0	46.30	1.7	36	1.192
5	100.0	46.07	1.8	22	1.021
4	99.8	46.41	1.8	15	0.940
3.8	99.9	43.37	2.1	13	0.920
3.55	99.9	40.27	2.4	4	0.847
3.3	99.9	35.20	2.8	8	0.920
3.05	100.0	30.36	3.7	10	0.935
2.8	99.9	23.71	5.3	9	0.906
2.45	100.0	16.07	8.6	6	0.865
2.2	100.0	10.88	13.7	3	0.836
1.95	98.0	5.68	28.8	3	0.791
					
Total	99.3	19.08	9.0	6	0.867

† Anomal. Corr. is the mean correlation factor between two random subsets of anomalous intensity differences.

‡ SigAno = [|*F*_(+)_ *F*_()_|/].
